# Eumycetoma Caused by Acremonium Species in a Farmer From Western Guatemala: A Case Report

**DOI:** 10.7759/cureus.107711

**Published:** 2026-04-25

**Authors:** John D Jaar, Allison R Serrano, Garbiñe Riley, Helga M Sarti, Liliana Acevedo

**Affiliations:** 1 Department of Dermatology, Instituto de Dermatología y Cirugía de Piel (INDERMA), Guatemala City, GTM; 2 Derpartment of Dermatology, Instituto de Dermatología y Cirugía de Piel (INDERMA), Guatemala City, GTM; 3 Department of Dermatopathology, Instituto de Dermatología y Cirugía de Piel (INDERMA), Guatemala City, GTM; 4 Department of Medical Mycology, Instituto de Dermatología y Cirugía de Piel (INDERMA), Guatemala City, GTM

**Keywords:** acremonium, eumycetoma, fungal infection, mycetoma, subcutaneous infection

## Abstract

Eumycetoma is a chronic subcutaneous infection caused by filamentous fungi and is clinically characterized by swelling, nodules, sinus tracts, and grain discharge. *Acremonium* species are rare opportunistic fungal pathogens implicated in eumycetoma, with few reports in the dermatologic literature. This report describes a 64-year-old male farmer from rural western Guatemala with a 14-year history of chronic dermatosis localized to the right foot. Clinical examination identified nodular lesions, crusting, and edema, consistent with mycetoma. Diagnosis was confirmed through mycological studies and morphological identification of *Acremonium *species.

## Introduction

Mycetoma is a chronic granulomatous infection of the skin and subcutaneous tissue caused by bacteria (actinomycetoma) or filamentous fungi (eumycetoma) and is endemic in tropical and subtropical regions [1,2]. Its classic clinical presentation is characterized by a triad of swelling, sinus tract formation, and granular discharge [3]. In Mesoamerica, eumycetoma is less frequently reported than actinomycetoma [1,4].

*Acremonium* species are hyaline, saprophytic filamentous fungi ubiquitous in soil and decaying organic matter, comprising approximately 150 described species [5,6]. Human infection typically results from traumatic inoculation of contaminated environmental material into the skin, most commonly affecting the feet of individuals with sustained occupational outdoor exposure, such as agricultural workers who frequently walk barefoot in endemic regions [7]. Although traditionally regarded as environmental contaminants, certain *Acremonium *species have emerged as rare opportunistic pathogens that can cause eumycetoma [5,6]. Diagnosis poses significant challenges: the clinical presentation may closely mimic other chronic infectious or inflammatory dermatoses; grain discharge may be minimal or absent in some cases; and definitive species-level identification often requires specialized mycological or molecular techniques that are not routinely available in resource-limited settings [3,8,9].

This case illustrates a rare and prolonged presentation of eumycetoma in a rural setting, characterized by delayed diagnosis, limited access to medical care, and interruption of treatment.

## Case presentation

**Figure 1:**
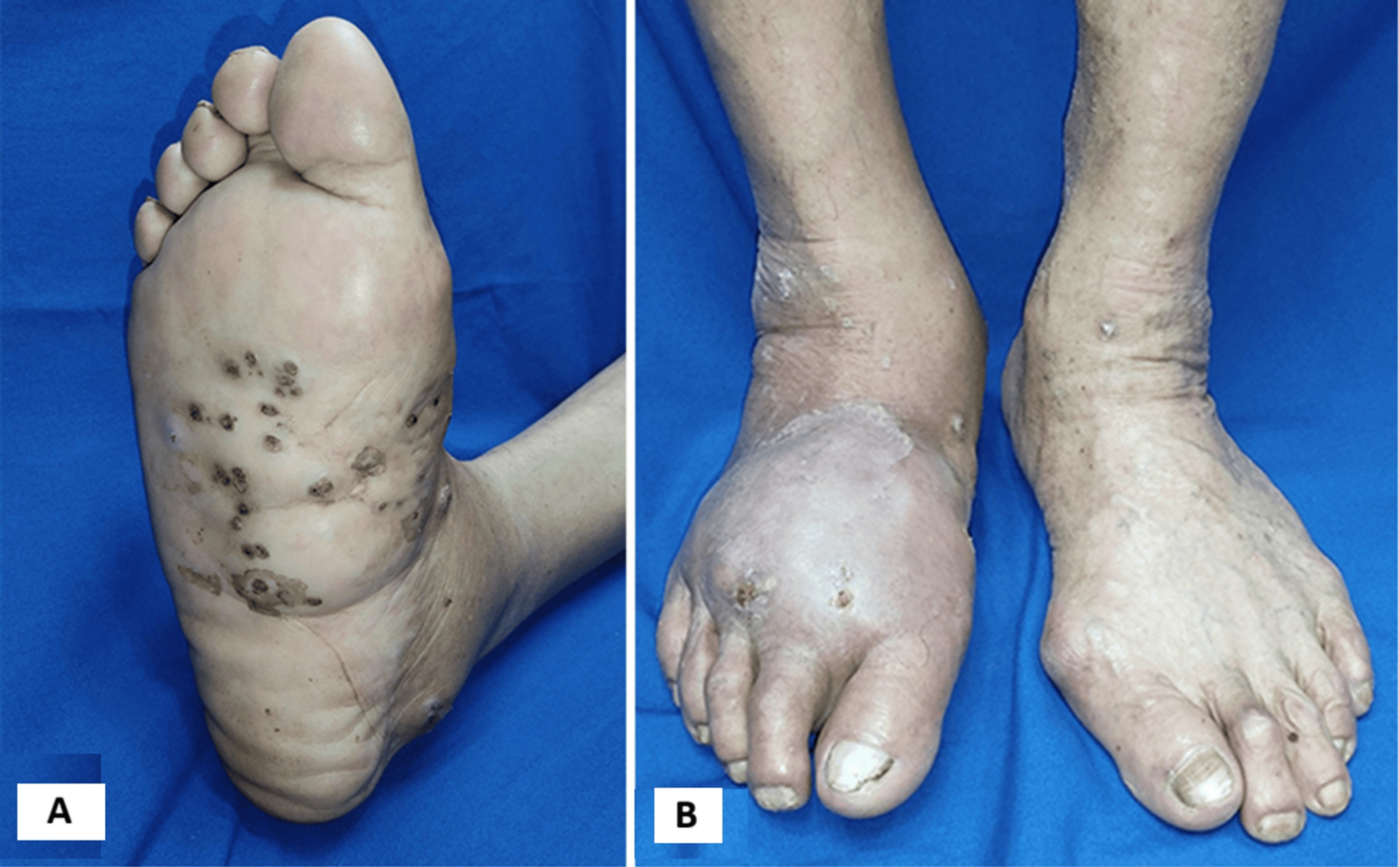
Initial clinical presentation of the right foot (A) Plantar view showing multiple nodular and crusted lesions with indurated borders. Note the diffuse erythema and the presence of sinus tract openings, which, together with nodular swelling and grain discharge, constitute the classic clinical triad of mycetoma [3,7]. (B) Lateral view demonstrating significant soft tissue edema and reduced mobility of the foot. The extent of swelling reflects the chronic, progressive nature of the infection and its impact on ambulation.

A 64-year-old male farmer from rural western Guatemala presented with a 14-year history of a chronic lesion on the right foot. The lesion began as a small papule of unknown origin and gradually progressed to multiple nodular, crusted, and ulcerated lesions. These were associated with pruritus, burning sensation, pain, and persistent inflammation, ultimately resulting in impaired ambulation (Figure 1).

The patient reported using homemade remedies and over-the-counter combination creams, neither of which resulted in clinical improvement. He had previously received a 17-day course of oral terbinafine without response. His medical history was significant only for arterial hypertension, for which he was not receiving pharmacological treatment at the time of presentation.

Dermatological examination revealed chronic dermatosis localized to an edematous right foot, characterized by polymorphic lesions. Multiple nodules, approximately 1 cm in diameter, were observed. These nodules were grayish to yellowish, with some ulcerated and others covered by honey-colored or whitish crusts. The lesions exhibited a rough, indurated surface with regular borders. Additionally, an edematous plaque involving both the dorsal and plantar surfaces of the foot, measuring up to 10 cm in diameter, was present. This plaque appeared erythematous, shiny, and firm, with poorly defined borders and superimposed nodules on xerotic skin. Plain radiographs of the foot revealed no lytic lesions or evidence of bone involvement, which argued against advanced osteomyelitis and supported a diagnosis confined to soft tissue at this stage.

The chronic, progressive, unilateral foot lesion with nodules, sinus tracts, and grain discharge in a rural agricultural worker raised the differential diagnosis of eumycetoma, actinomycetoma, botryomycosis, and chromoblastomycosis [9]. The absence of response to prior antifungal therapy with terbinafine and the clinical morphology were most consistent with eumycetoma caused by a non-dermatophytic mold.

**Figure 2:**
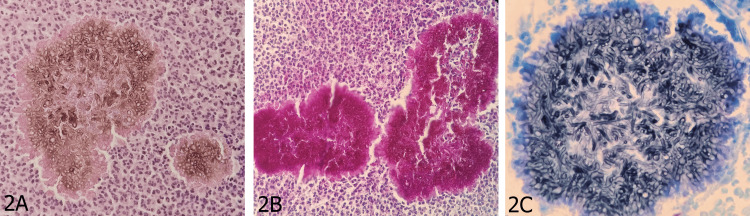
Histopathological findings (A) Hematoxylin and eosin (H&E) staining showing compact fungal grains - dense aggregates of hyphae - embedded within the dermis and surrounded by a mixed inflammatory infiltrate of neutrophils and lymphocytes. The identification of grains on histopathology is the pathological hallmark of mycetoma, distinguishing it from other deep fungal infections [3,11]. (B) Periodic acid-Schiff (PAS) staining highlighting a fungal grain with intense magenta positivity. PAS selectively stains fungal polysaccharides, confirming the fungal nature of the organisms and delineating hyphal architecture within the grain [11]. (C) Kinyoun staining demonstrating a large grain with intense blue staining, surrounded by granulomatous inflammation. The absence of acid-fast bacilli in this preparation supports a fungal rather than bacterial etiology [11].

Based on the clinical findings, a diagnosis of mycetoma was suspected, and samples were obtained for skin biopsy and mycological studies. Histopathological examination with hematoxylin and eosin staining (Figure 2, Panel A) demonstrated epidermis with basket-weave stratum corneum and the presence of neutrophilic abscesses. In the superficial reticular dermis, grains of varying sizes composed of fungal structures were identified, surrounded by a mixed inflammatory infiltrate of neutrophils and lymphocytes extending into the deep reticular dermis. The grains - compact aggregates of hyphae that are the hallmark pathological feature of eumycetoma - exhibited well-defined contours and dense architecture, embedded within surrounding granulomatous tissue [3,10,11]. The presence of grains on histopathology, combined with the absence of filamentous bacterial structures, confirmed a fungal rather than bacterial etiology, effectively ruling out actinomycetoma. Periodic acid-Schiff staining (Figure 2, Panel B) highlighted an irregularly contoured grain with intense magenta positivity, composed of compact hyphae morphologically enclosed within an inflammatory granuloma, further confirming the fungal nature of the organisms. Kinyoun staining (Figure 2, Panel C) revealed a large grain composed of aggregated fungal structures, which showed intense blue staining and was surrounded by a granulomatous inflammatory reaction; the absence of acid-fast bacilli on this stain further helped exclude mycobacterial infection as an alternative diagnosis.

**Figure 3:**
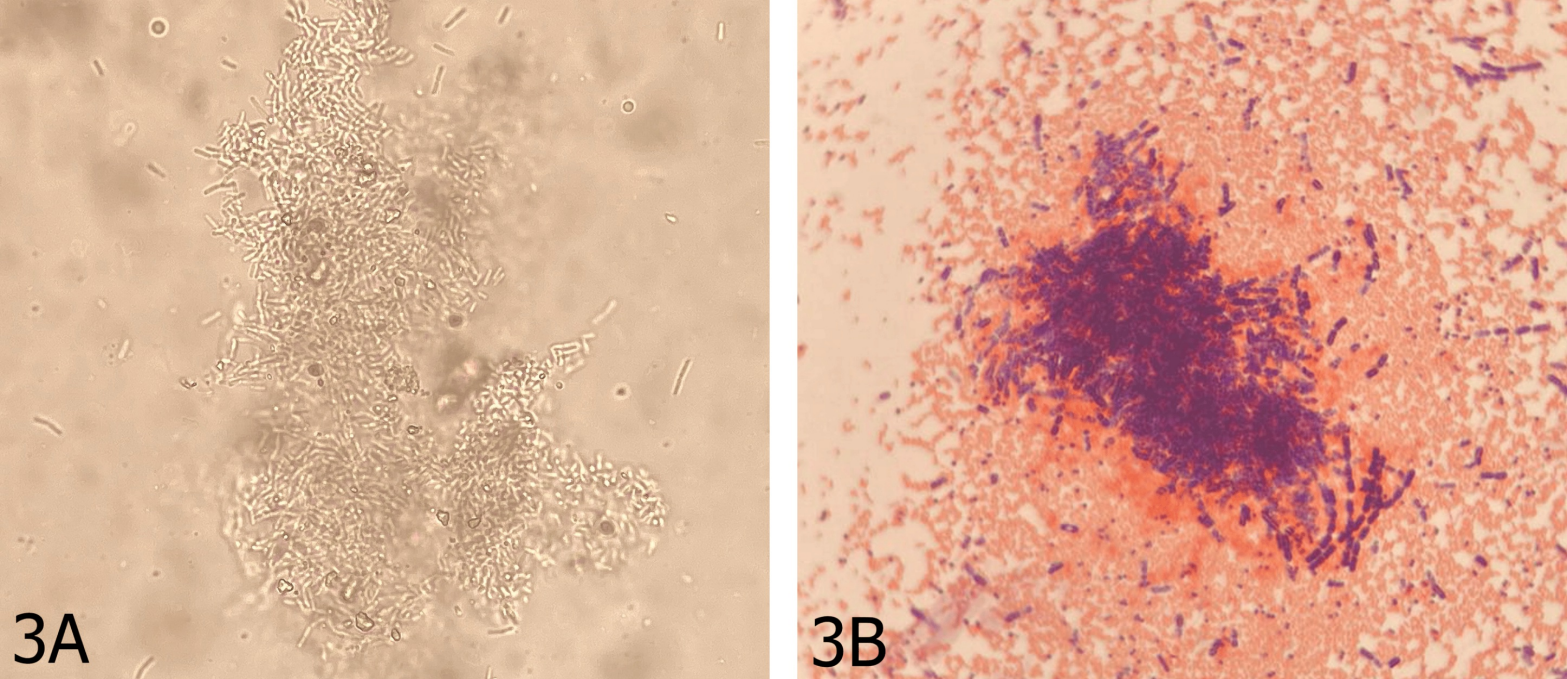
Direct mycological examination of the lesion (A) Potassium hydroxide (KOH) preparation revealing abundant septate hyphae consistent with fungal elements. KOH dissolves keratin and host tissue debris, allowing direct visualization of fungal structures [8]. (B) Gram staining demonstrating a fungal grain composed of Gram-positive septate hyphae embedded within granulomatous material. The thick, true hyphae seen in this image are characteristic of eumycetoma and differ from the thin, branching filaments observed in actinomycetoma [10].

Direct examination with potassium hydroxide (KOH) preparation (Figure 3, Panel A) revealed abundant septate hyphae consistent with fungal elements. Gram staining (Figure 3, Panel B) demonstrated a fungal grain composed of Gram-positive septate hyphae embedded within abundant granulomatous material, a finding that further distinguishes this from actinomycetoma, in which filamentous bacterial elements would appear as thin, branching Gram-positive filaments without true hyphae.

Clinical and mycological samples - including scrapings from sinus tract discharge and tissue fragments from nodular lesions - were inoculated onto Sabouraud dextrose agar (SDA), with and without cycloheximide, and incubated at 25°C and 37°C [5,6]. Cultures yielded slow-growing, white to pale-gray colonies with a powdery surface. Microscopic examination of the cultures revealed hyaline, septate hyphae bearing characteristic solitary phialides with ellipsoidal to cylindrical conidia arranged in slimy clusters, morphologically consistent with *Acremonium *species [5,6]. Species-level identification by molecular methods (internal transcribed spacer (ITS) sequencing or MALDI-TOF mass spectrometry) was not available at the treating institution. Fungal culture thus identified *Acremonium* species as the etiologic agent, confirming the diagnosis of eumycetoma. These microbiological findings were consistent with the clinical presentation: the chronic indolent course, pedal localization, and whitish grain morphology on direct examination are characteristic features of *Acremonium*-associated eumycetoma [4,5,10,11].

Treatment was initiated with oral itraconazole 200 mg daily, along with ciprofloxacin for 21 days, due to clinical signs of a secondary bacterial infection. As a local adjunctive measure, topical aluminum sulfate dressings were applied. After completion of antibiotic therapy, antifungal treatment was continued as monotherapy.

**Figure 4:**
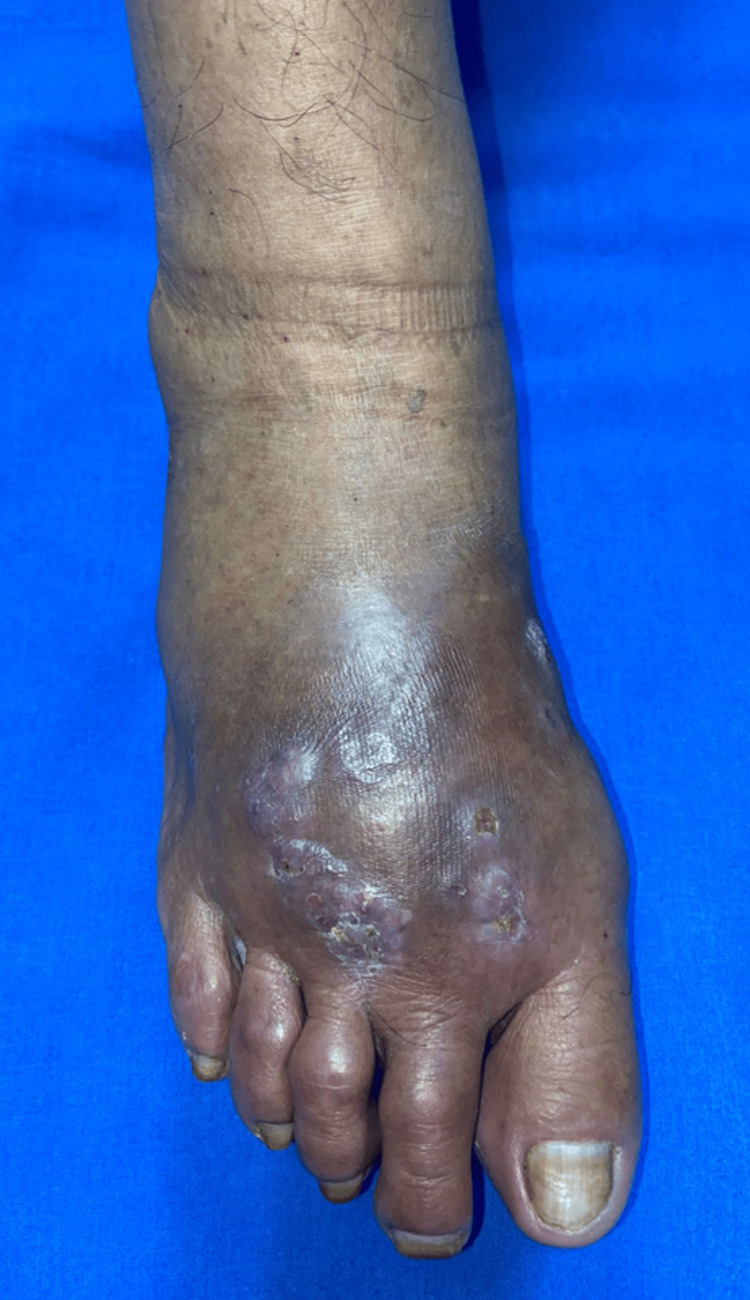
Clinical evolution after antifungal treatment This figure shows the clinical appearance of the affected foot after two months of oral itraconazole therapy, demonstrating decreased soft tissue edema, reduction in the size and number of nodular lesions, and partial resolution of crusts with areas of desquamation. These findings indicate a partial but clinically meaningful response to antifungal therapy. Complete resolution was not achieved within the observation period, consistent with the typically prolonged treatment course required in eumycetoma [12].

After eight weeks of treatment, a progressive reduction in inflammation and partial clinical improvement were observed, including decreased edema and early resolution of nodular lesions (Figure 4). Despite these improvements, the patient discontinued follow-up. Access to the dermatology center was facilitated by a neighbor who provided transportation to the capital city. No further contact with the patient has been established.

## Discussion

The genus *Acremonium* comprises hyaline filamentous fungi that are saprophytic in nature and commonly found in soil and decaying vegetation. Although traditionally regarded as environmental contaminants, several *Acremonium* species have been increasingly recognized as opportunistic human pathogens, including rare cases of eumycetoma [1,2].

Eumycetoma caused by *Acremonium* species is uncommon when compared with more prevalent etiologic agents such as *Madurella mycetomatis* or Fusarium species [3]. Reported cases have been described mainly in Latin America and Asia, commonly involving pedal forms of mycetoma, with mycological isolation of species such as *Acremonium falciforme *[4,10]. These reports demonstrate the geographic and clinical variability of *Acremonium*-associated eumycetoma.

Clinically, *Acremonium*-related mycetoma commonly follows a chronic course characterized by nodular lesions, sinus tract formation, and occasional discharge of whitish or yellowish grains. However, grain extrusion may be minimal or absent in some cases, complicating clinical recognition and delaying diagnosis. In this context, mycological culture remains the diagnostic gold standard, although advanced techniques such as molecular identification or matrix-assisted laser desorption/ionization time-of-flight (MALDI-TOF) mass spectrometry are recommended for exact species-level identification when available [8,11].

From a therapeutic standpoint, *Acremonium* species display variable antifungal susceptibility patterns. In vitro studies have demonstrated resistance to fluconazole and ketoconazole, while itraconazole, voriconazole, and posaconazole generally show better antifungal activity [5,12]. Nonetheless, medical treatment often requires prolonged therapy and close clinical monitoring. In advanced cases, antifungal therapy may need to be combined with surgical intervention to achieve optimal outcomes [13,14].

Managing mycetoma should involve clinical, microbiological, and imaging findings. Some authors have suggested combined treatment plans and decision-making guides, but for *Acremonium* species, there is not enough evidence yet to make standard recommendations [15,16].

Beyond microbiological and therapeutic considerations, chronic mycetoma has a substantial impact on patient quality of life. Persistent pain, impaired mobility, and functional disability negatively affect occupational performance and social relationships, particularly among agricultural workers reliant on physical labor. In this case, logistical and economic barriers limited access to specialized care, with transportation to the capital city dependent on community support. These constraints led to treatment discontinuation and loss to follow-up. The challenges described reflect broader systemic issues in health care accessibility in rural and resource-limited settings, where social determinants such as poverty, geographic isolation, and limited infrastructure often impede timely diagnosis, adherence to therapy, and long-term disease management [7,9].

## Conclusions

This case highlights the diagnostic and therapeutic challenges posed by eumycetoma caused by rare pathogens, such as *Acremonium *species, particularly in resource-limited rural settings where access to specialized care is restricted. Early clinical recognition of the characteristic triad of swelling, sinus tract formation, and grain discharge remains essential to initiate timely investigation and treatment. However, limitations of this report must be acknowledged: definitive species-level identification was not achieved due to the unavailability of molecular diagnostic techniques at the treating institution, and the patient was lost to follow-up after eight weeks, leaving the long-term clinical outcome undocumented. Conclusions regarding treatment efficacy must therefore be interpreted with caution.

Reporting such cases contributes to the growing literature on *Acremonium*-associated eumycetoma in Latin America and underscores the need for integrated public health strategies, including community education, improved access to diagnostic resources, and structured long-term follow-up programs. Continuity of care remains fundamental to improving prognosis in chronic neglected tropical infections such as mycetoma.
